# Integrative Multiomics Analysis Reveals a Cancer Stem Cell–Driven Prognostic Signature and Nominates Belinostat for Targeted Therapy in Hepatocellular Carcinoma

**DOI:** 10.1155/sci/7388629

**Published:** 2026-08-02

**Authors:** Yang Zi, Ying Zhang, Jun Wu, Jingjing Xie, Xuehua Yan

**Affiliations:** ^1^ Department of Intervention, Inner Mongolia People’s Hospital, Hohhot City, Inner Mongolia Autonomous Region, China, nmgyy.cn; ^2^ Department of Hepatology, Integrated Traditional Chinese and Western Medicine, Lanzhou Second People’s Hospital, Lanzhou City, Gansu Province, China; ^3^ Department of Radiology, Lanzhou Second People’s Hospital, Lanzhou, City, Gansu Province, China; ^4^ Department of Medical Science, University of Technology Sydney, Sydney, New South Wales, Australia, uts.edu.au

**Keywords:** belinostat, cancer stem cells, drug repositioning, hepatocellular carcinoma, prognostic signature

## Abstract

**Background:**

Hepatocellular carcinoma (HCC) suffers from a poor prognosis largely due to its profound molecular heterogeneity and high frequency of relapse, challenges that are closely linked to the biology of cancer stem cells (CSCs) and a lack of effective stemness‐related prognostic biomarkers. Identifying CSC–related prognostic biomarkers and therapeutic targets is critical for improving patient outcomes.

**Methods:**

We integrated differential expression analysis, weighted gene co‐expression network analysis (WGCNA), and CSC gene databases to identify core prognostic genes driven by stemness mechanisms. A robust prognostic model was developed and validated using multiple machine learning algorithms across TCGA and Gene Expression Omnibus (GEO) cohorts. The clinical relevance of the signature was assessed via receiver operating characteristic curve (ROC) curves, survival analysis, and association with tumor stage. Single‐cell RNA sequencing (scRNA‐seq) and computational drug repositioning coupled with molecular docking were employed to explore mechanistic insights and therapeutic candidates.

**Results:**

Intersection analysis identified 12 core genes enriched in CSC–associated pathways. The optimal CoxBoost model demonstrated superior predictive performance for overall survival (OS) in internal, external, and meta‐analyses. The signature’s single‐sample GSEA (ssGSEA) score exhibited high diagnostic accuracy, correlated with advanced tumor stage, and enabled effective risk stratification. Single‐cell analysis revealed DARS2 enrichment in M1 macrophages, suggesting a role for CSCs in modulating the tumor immune microenvironment. The histone deacetylase (HDAC) inhibitor belinostat was prioritized via Drug Signature Database (DSigDB) screening and validated by molecular docking as a candidate for targeting the CSC–related signature.

**Conclusion:**

This study establishes a novel CSC–associated gene signature for diagnosis and prognosis in HCC and nominates belinostat as a repurposing candidate for targeting stemness‐related pathways, offering a promising strategy for personalized therapy.

## 1. Introduction

Hepatocellular carcinoma (HCC) represents a major global health burden, ranking as a leading cause of cancer‐related mortality worldwide [1–3]. Its pathogenesis is complex, driven by heterogeneous genetic and epigenetic alterations, which contribute to aggressive tumor behavior, frequent recurrence, and limited therapeutic efficacy, particularly in advanced stages [4]. Current first‐line targeted therapies, such as sorafenib and lenvatinib, offer only modest survival benefits, with response rates below 30% and nearly universal acquired resistance, highlighting an urgent unmet clinical need. Despite advances in surveillance and treatment, the prognosis for HCC patients remains unsatisfactory, underscoring the urgent need for reliable prognostic biomarkers to guide risk stratification and personalized treatment decisions [5, 6].

The emergence of high‐throughput transcriptomic technologies and bioinformatics has enabled the systematic discovery of molecular signatures in cancer [7–10]. However, many existing HCC prognostic models suffer from limitations, including a lack of robust validation, insufficient biological context, and poor clinical translatability. Crucially, most current models fail to explicitly incorporate the cancer stem cell (CSC) perspective, often overlooking the core driver role of stemness in tumor initiation, progression, and therapy resistance. A particular challenge lies in distinguishing core driver genes from passenger alterations in the vast landscape of transcriptomic changes [11–13].

To address these challenges, we employed an integrated multistep bioinformatics approach. First, we mapped the global transcriptomic alterations in HCC through differential expression analysis. Second, we utilized weighted gene co‐expression network analysis (WGCNA) to identify modules of co‐expressed genes that are highly correlated with clinical traits, moving beyond individual genes to capture functionally coordinated networks. Given the critical role of CSCs in HCC initiation, progression, and therapy resistance, we integrated a curated CSC gene set. The intersection of these three dimensions—differential expression, co‐expression networks, and stemness biology—was designed to filter for a highly relevant and robust gene set, representing a novel integrative strategy for biomarker discovery in HCC.

The primary objectives of this study were as follows: (1) to identify a core set of HCC prognostic genes through this integrative pipeline; (2) to construct and rigorously validate a prognostic model using state‐of‐the‐art machine learning algorithms across multiple independent cohorts; (3) to comprehensively evaluate the diagnostic value and clinical relevance of the signature; (4) to explore its therapeutic implications through drug repositioning and computational validation. This systematic strategy aims to deliver a biologically grounded and clinically applicable tool for improving HCC management.

## 2. Materials and Methods

### 2.1. Data Acquisition

The data regarding gene expression were acquired from the public database of the NCBI Gene Expression Omnibus (GEO). The liver cancer dataset GSE101685 was used for differential analysis. Tumor‐related stem cell genes were obtained from the GENECARDS database.

### 2.2. Differential Expression Analysis

The analysis of differential gene expression was performed utilizing the “limma” package within the R programming environment. Genes exhibiting a false discovery rate (FDR)–adjusted *p*‐value of less than 0.05 and an absolute log fold change (abs(log FC)) exceeding 0.585 were classified as differentially expressed genes (DEGs). To facilitate the visualization of these DEGs, heatmaps and volcano plots were created employing the “pheatmap” and “ggplot2” packages, respectively.

### 2.3. Weighted Correlation Network Analysis (WGCNA)

To investigate the co‐expression dynamics of genes and their associations with phenotypic traits, we employed the “WGCNA” package within the R programming environment to develop a gene co‐expression network. Following the analysis of dendrograms, we excluded samples from our dataset that exhibited atypical characteristics. We focused on the top 5000 genes, which were defined by a median absolute deviation (MAD) greater than one. The construction of a similarity matrix was achieved by calculating the correlation coefficients for each gene pair. To facilitate the formation of a network adhering to the scale‐free topology, an appropriate soft threshold was selected to convert the similarity matrix into an adjacency matrix. Subsequently, we generated a topological overlap matrix (TOM) to assess the average connectivity of each gene. Utilizing the dynamic tree cutting method, we categorized genes with analogous expression patterns into separate modules, guided by parameters such as minModuleSize and mergeCutHeight within the blockwiseModules function. Each module was assigned a unique color, while genes categorized in gray modules were those that did not fit into any specific module. To characterize the gene expression profile of each module, we computed a principal component referred to as the module eigengene (ME). To evaluate the relationship between the modules and phenotypic traits, we analyzed the MEs. The module demonstrating a significant absolute correlation coefficient was designated as the primary module for subsequent analyses. Module membership (MM) is defined as the correlation coefficient that quantifies the relationship between a gene’s expression value and the ME of its respective module, indicating the strength of the correlation between the gene and the module. Gene significance (GS) represents the correlation coefficient between a gene’s expression value and a phenotype, thereby illustrating the relationship between genes and phenotypic traits.

### 2.4. Consensus Prognosis Model

In line with recent studies integrating machine learning and multiomics data to identify prognostic molecular signatures, we utilized a range of modeling techniques, including Lasso regression, elastic net, ridge regression, stepwise Cox regression, and CoxBoost [14]. The Lasso regression was executed via the glmnet package, with the family parameter designated as Cox and the alpha parameter established at 1. The cv.glmnet function was employed to perform tenfold cross‐validation to determine the optimal *λ* value. Subsequently, we extracted the model coefficients and feature names associated with the optimal *λ* value from the training outcomes, filtering out nonzero coefficients along with their corresponding gene identifiers. Both elastic net and ridge regression were also implemented using the glmnet package, where the alpha parameter for elastic net varies between 0 and 1, while for ridge regression, it is fixed at 0. The stepwise Cox regression commenced by constructing a multivariate Cox regression model with the coxph function, followed by a stepwise regression analysis using the stepAIC function, incorporating both forward and backward selection methods. For the CoxBoost model, we initially optimized the penalty parameters through the optimCoxBoostPenalty function, followed by cross‐validation using the cv.CoxBoost function to ascertain the optimal number of steps. Ultimately, the CoxBoost model was developed utilizing the CoxBoost function alongside the identified optimal number of steps and penalty parameters. The model coefficients and corresponding genes were retrievable via the coef function or from the regression coefficient slot of the respective model.

To derive the risk score for each sample, we calculated the linear combination of gene expression data and model coefficients. The efficacy of the prognostic model was assessed using the receiver operating characteristic curve (ROC) and the area under the curve (AUC) as evaluation metrics. The timeROC package facilitated the calculation of AUC values at various time intervals (1, 3, and 5 years) to appraise the performance of multiple prognostic models. Ultimately, we employed the ComplexHeatmap package to visualize the average AUC values across the specified time points. Additionally, we conducted a univariate Cox analysis using the coxph function to compute the hazard ratio (HR) values of the risk score derived from the top‐performing algorithm across different datasets. To further substantiate the reliability of our findings, we performed a meta‐analysis of the univariate Cox survival analysis results employing the inverse variance method, with the logarithmic HR value serving as the primary measurement parameter to evaluate the model’s prognostic significance. Finally, we executed Kaplan–Meier survival analysis using the survival package and identified the optimal cutoff values for high‐risk and low‐risk groups through the survminer package, ensuring that the minimum ratio between these groups did not fall below 0.3. The log‐rank test was carried out utilizing the survfit function to assess the significance of differences between the high‐risk and low‐risk cohorts.

### 2.5. Single‐Cell Analysis

To explore the expression and cell type specificity of 12 core prognostic genes at single‐cell resolution, we analyzed liver cancer‐related single‐cell RNA sequencing (scRNA‐seq) data from public databases. Data preprocessing includes quality control filtering, normalization, and batch effect correction. We adopted the graph theory‐based clustering method to cluster the cells and annotated the cell subpopulations using classical cell lineage marker genes (such as CD3E for T cells, CD19 for B cells, CD68 for macrophages, etc.).

Subsequently, we extracted the expression levels of 12 candidate genes in each cell and drew violin plots to visualize their probability density distribution, median expression, and numerical range in different cell subpopulations. This analysis aims to identify genes that are specifically highly expressed in particular cell types, especially those related to the tumor stem cell microenvironment, thereby providing clues for interpreting the potential cytological mechanisms of prognostic characteristics.

### 2.6. Drug Repurposing via Drug Signature Database (DSigDB) for Prognostic Signature

To translate our HCC prognostic signature into actionable therapeutic targets, we utilized the DSigDB, a curated resource linking drugs/compounds to target gene sets for gene set enrichment analysis (GSEA). DSigDB (version 1.0, accessed via http://tanlab.ucdenver.edu/DSigDB) curates 22,527 gene sets encompassing 17,389 unique compounds and 19,531 genes, enabling systematic integration of drug activity with transcriptomic profiles [15]. We performed a signature‐based drug screening by querying DSigDB with our prognostic signature. This analysis identified belinostat (CTD:00004313), an FDA–approved histone deacetylase (HDAC) inhibitor, suggesting mechanistic relevance to our prognostic gene network.

### 2.7. Molecular Docking

Molecular docking serves as a predictive technique to ascertain the optimal orientation of a ligand when it interacts with a receptor, which may include biomolecules such as RNA or enzymes. In the present investigation, we employed a semiflexible docking approach to facilitate the formation of a stable complex. This methodology is pivotal for elucidating the underlying mechanisms of action or for the identification of lead compounds, thereby establishing it as a cornerstone in structure‐based drug design. The compound Belinostat (PubChem CID: 6918638) was docked with the proteins AURKA (Uniprot ID: O14965) and BIRC5 (Uniprot ID: not specified) utilizing AutoDock Vina version 1.1.2. Additionally, molecular docking was executed with the proteins CDK1 (Uniprot ID: P06493), CDK4 (Uniprot ID: P11802), and PARP1 (Uniprot ID: P09874). The structural files of the compounds were obtained from the PubChem database (https://pubchem.ncbi.nlm.nih.gov/). Furthermore, Chemdraw 20.0 was employed to construct three‐dimensional models of the compounds and to perform energy minimization. The protein models were sourced from the AlphaFold Protein Structure Database (https://alphafold.ebi.ac.uk/).

### 2.8. Statistical Analysis

All analyses were performed in R software. The *t*‐test and Mann–Whitney *U*‐test were chosen depending on the conformity of the data to the normal distribution. Typically, significance is defined as *p* < 0.05.

## 3. Result

### 3.1. Differential Expression Analysis Revealed Significant Transcriptome Differences Between Liver Cancer and Control Tissues

**Figure 1:**
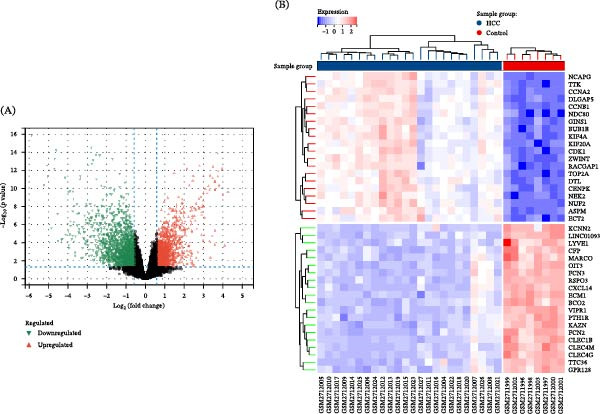
Differential expression analysis revealed significant transcriptome differences between liver cancer and control tissues. (A) The volcano plot shows the differentially expressed genes between liver cancer tissues and control tissues. (B) Heatmap of differentially expressed genes.

Significant transcriptome differences were observed between liver cancer and control tissues, with a total of 2933 DEGs identified (Figure 1A). Green triangles denoted downregulated genes, red triangles upregulated genes, and black dots nonsignificant genes (Figure 1A). Figure 1B (heatmap) further displays these DEGs’ expression profiles across samples—rows represent genes, columns represent samples, and blue‐to‐red gradients indicate low‐to‐high expression. Sample clustering clearly separated HCC from control groups, while gene clustering grouped DEGs into coordinated expression clusters, reflecting potential functional coherence. The sheer number and magnitude of these changes underscore the profound transcriptomic reprogramming that characterizes HCC, setting the stage for identifying key drivers within this complex landscape.

### 3.2. WGCNA Identifies the Core Modules Related to the Clinical Traits of Liver Cancer

**Figure 2:**
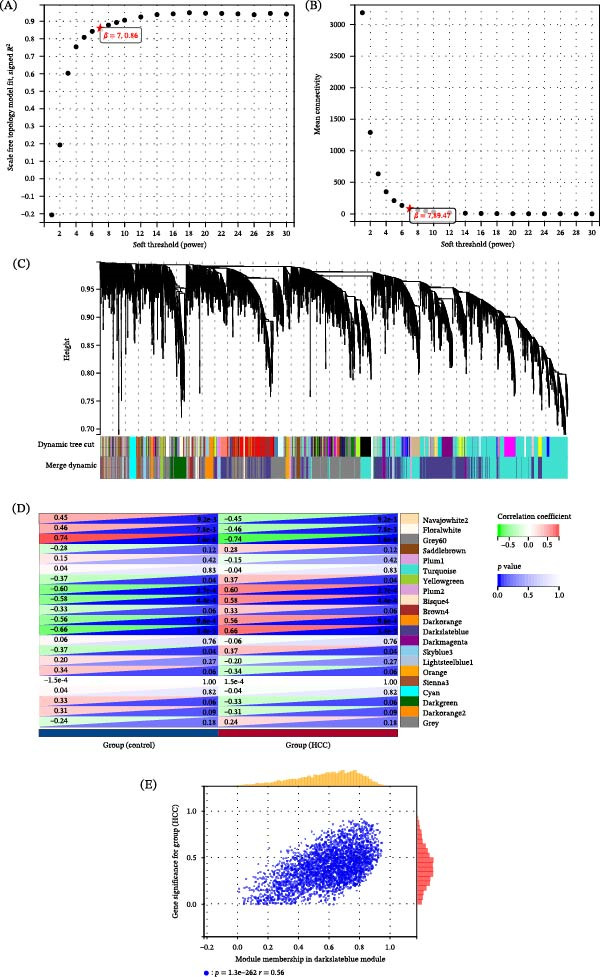
Weighted gene co‐expression network analysis identifies the core modules related to the clinical traits of liver cancer. (A) Soft threshold screening graph. (B) Average connectivity graph. (C) Gene clustering tree diagram and module allocation. (D) Heatmap of module–trait relationship. The correlations between each co‐expressed module and clinical traits were demonstrated. The darkslateblue module showed the strongest positive correlation with the target clinical trait (*R* = 0.66). Scatter plot of module membership degree and gene significance of genes in the (E) darkslateblue module.

Having established that HCC is characterized by widespread transcriptomic alterations (2933 DEGs), we next sought to move beyond individual genes and identify co‑expression modules that operate as functional networks correlated with clinical traits. Therefore, a robust co‑expression network module strongly correlated with HCC clinical traits was identified using the WGCNA. A robust co‐expression network module strongly correlated with HCC clinical traits was identified using WGCNA. As shown in Figure 2A,B, a soft threshold power of *β* = 7 achieved a scale‐free topology fit of 0.86 (Figure 2A) and a mean connectivity of approximately 789.47 (Figure 2B), ensuring a robust co‐expression network. Figure 2C (dendrogram + module color bar) clusters genes by expression similarity, with colored bars assigning genes to distinct modules (e.g., darkslateblue). Figure 2D (module‐trait heatmap) revealed module‐phenotype correlations, with the darkslateblue module showing the strongest positive correlation (*R* = 0.66) with the target clinical trait. Figure 2E (MM vs. GS scatter plot) validated this association: for darkslateblue module genes, MM positively correlated with GS for KRT88 (*p* = 1.3e–05). Notably, the darkslateblue module contained 419 genes, representing a core co‐expressed subnetwork functionally tied to HCC pathogenesis. The strong positive correlation of this module with clinical traits suggests that these genes operate in a coordinated manner to drive key malignant behaviors, providing a biologically relevant context beyond a simple list of individual genes.

### 3.3. Screening of Core Genes Related to Prognostic Tumor Stem Cells and Evaluation of Prognostic Model Algorithms

**Figure 3:**
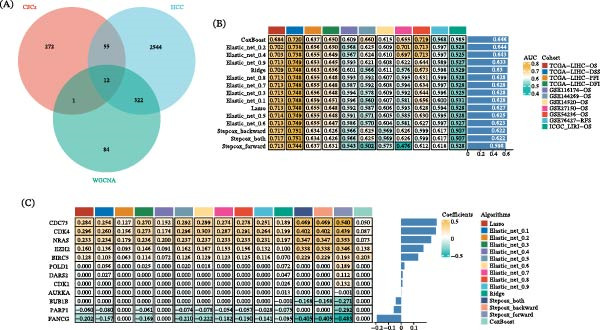
Screening of core genes related to prognostic tumor stem cells and evaluation of prognostic model algorithms. (A) Venn diagrams show the intersection of genes related to cancer stem cells, differentially expressed genes, and genes in the WGCNA module. A total of 12 candidate genes were identified from the intersection of the three groups of genes for the construction of the prognostic model. (B) Heatmaps of the average AUC values of prognostic models constructed by different algorithms at different queues and time points. The algorithm is sorted from high to low by its average AUC value. The CoxBoost algorithm demonstrates the highest prediction accuracy in most cases. (C) Coefficient heatmaps of 12 candidate genes in different algorithm models.

Twelve core genes intersecting CSC–related genes, DEGs, and WGCNA module genes were identified as optimal candidates for prognostic model construction (Figure 3A). Specifically, we constructed a Venn diagram (Figure 3A) to visualize the intersection of three gene sets: (1) 339 CSC–related genes retrieved from GENECARD; (2) DEGs identified via differential expression analysis; (3) genes derived from WGCNA (Table S1). The intersection of these three sets yielded 12 overlapping genes, which were selected as candidate genes for subsequent model construction. Figure 3B presents a heatmap of the average AUC values for prognostic models built using 12 distinct algorithms (e.g., CoxBoost, Elastic_net variants, Ridge, and Stepcox series) across multiple survival time points (1‐, 3‐, and 5‐year) and cohorts (e.g., TCGA‐LIHC‐OS and GSE14520‐OS). Algorithms were ranked from top to bottom by their mean AUC across all datasets, enabling the identification of the most consistently high‐performing algorithm. Notably, the CoxBoost algorithm exhibited the darkest red shading (highest AUC values) across most time points and cohorts, indicating its superior predictive accuracy for patient survival compared to other algorithms (e.g., Elastic_net, Ridge, and Stepcox variants). Figure 3C visualizes gene coefficients in prognostic models generated by the 12 algorithms. Coefficients reflect a gene’s contribution to the model: a coefficient of 0 indicates that the gene was excluded by the algorithm, while nonzero values denote inclusion (with larger absolute values indicating greater contribution). The color scale (blue to red) corresponds to coefficient values ranging from −0.5 to 0.5. This heatmap elucidates the heterogeneity in gene prioritization across modeling strategies, informing subsequent interpretations of model biology. Collectively, these results validate the rationality of the 12‐gene candidate set, establish CoxBoost as the optimal algorithm for prognostic modeling, and characterize algorithm‐specific gene contributions, laying a foundation for the mechanistic and clinical validation of the prognostic model.

### 3.4. Single‐Cell Expression Profiles of 12 Genes in Multiple Immune/Hematopoietic Cell Types

**Figure 4:**
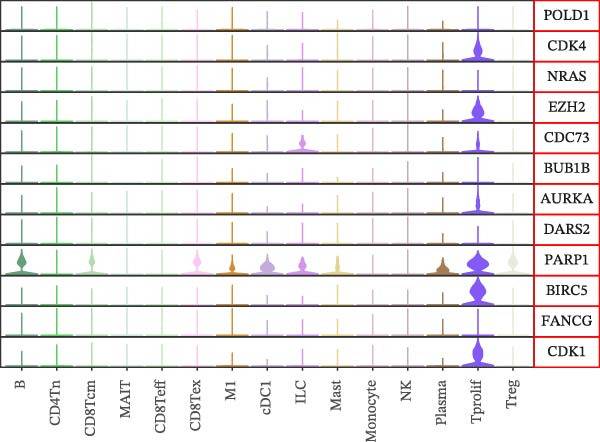
Violin plots of the expression and distribution of 12 candidate genes in 14 types of immune and hematopoietic cells. The *Y*‐axis represents the expression level and the *X*‐axis represents the cell type. DARS2 is specifically highly expressed in M1‐type macrophages. PARP1 shows broad‐spectrum expression. The expression levels of POLD1, NRAS and FANCG were generally low in all cell types.

Having established that CoxBoost was the optimal algorithm and that the 12‑gene set had prognostic utility at the bulk tissue level, we next asked whether these genes exhibit cell‑type‐specific expression patterns within the tumor microenvironment. To address this, we performed single‑cell RNA sequencing analysis. Cell‑type‐specific expression patterns of the 12 candidate genes were revealed by single‑cell transcriptomic analysis across 14 immune and hematopoietic cell types (Figure 4). Figure 4 presents the single‐cell expression profile characteristics of 12 candidate genes in 14 immune and hematopoietic source cell types. The probability density distribution, median, and numerical range of gene expression are visualized through violin plots (with cell types on the *X*‐axis). Covering B cells, naive CD4^+^T cells (CD4Tn), central memory CD8^+^T cells (CD8Tcm), muco‐associated constant T cells (MAIT), exhausted CD8^+^T cells (CD8Tex), M1‐type macrophages, Type I classical dendritic cells (cDC1), Type 2 innate lymphocytes (ILCs), mast cells Mast, monocytes, natural killer (NK) cells, plasma cells (plasma), proliferative T cells (Tprolif), and regulatory T cells (Treg). DARS2 only shows a specific expression in M1‐type macrophages. Considering the characteristic of “pro‐inflammatory polarization accompanied by metabolic reprogramming” of M1‐type macrophages, it is speculated that DARS2 may be involved in the metabolic regulation of macrophage polarization. The expression pattern of PARP1 shows a wide distribution across cell types. However, the expression levels of POLD1, NRAS, and FANCG were generally low in all cell types, and no significant cell type‐specific enrichment was shown.

### 3.5. Multicohort Meta‐Analysis of Prognostic Risk Scores and Survival Verification

**Figure 5:**
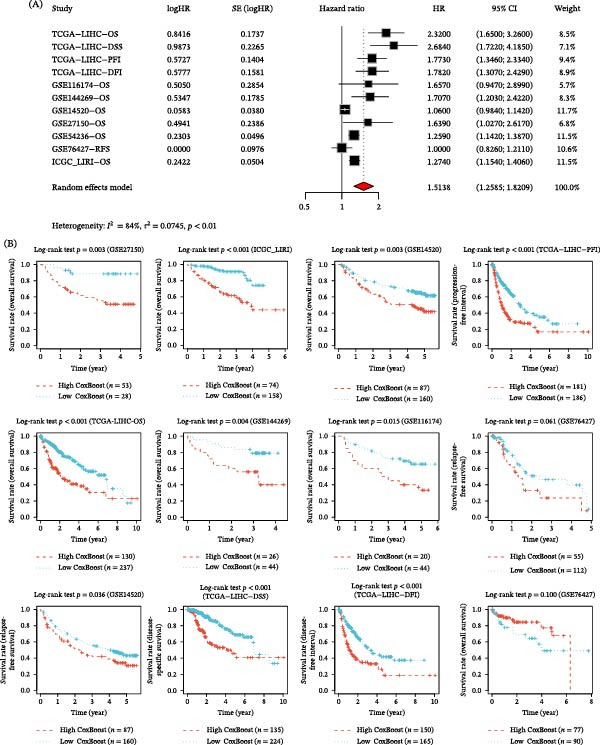
Meta‐analysis and survival validation of the prognostic risk score across cohorts. (A) A multicohort meta‐analysis forest plot based on the risk score of the CoxBoost model. The combined hazard ratio indicates that this risk score is a significant independent prognostic factor. (B) Kaplan–Meier survival curves of the high‐risk and low‐risk groups based on the optimal cutoff in the TCGA‐LIHC cohort. The overall survival period of patients in the high‐risk group was significantly shorter.

The single‑cell analysis revealed interesting patterns (e.g., DARS2 enrichment in M1 macrophages), but the core question remained whether the 12‑gene signature could consistently predict patient survival across independent cohorts. Therefore, we conducted a multicohort meta‑analysis of the prognostic risk score derived from the CoxBoost model. The prognostic risk score was confirmed as a significant independent prognostic factor across multiple independent cohorts (meta‐analysis, Figure 5A). To evaluate the prognostic model’s consistency across independent studies, we conducted a meta‐analysis using HRs with 95% confidence intervals (CIs) to quantify the standard error of HR (Figure 5A). This approach aggregated results from multiple cohorts, enhancing the statistical power and the reliability of the conclusions. The pooled analysis revealed that the risk score derived from our CoxBoost model served as a significant independent prognostic factor. This result confirms the model’s robust predictive performance and generalizability in diverse patient populations. Kaplan–Meier survival curves further validated the model’s clinical utility (Figure 5B). A log‐rank test demonstrated a statistically significant difference in survival outcomes between the two groups (*p* < 0.001): the high‐risk group exhibited markedly poorer overall survival (OS) compared to the low‐risk group. This finding directly correlates with the meta‐analysis results—our CoxBoost‐based model effectively stratifies patients by prognosis, underscoring its clinical relevance for personalized risk assessment and management.

### 3.6. The Expression, Diagnostic Value of Prognostic Gene Sets, and Their Association With Clinical Stages

**Figure 6:**
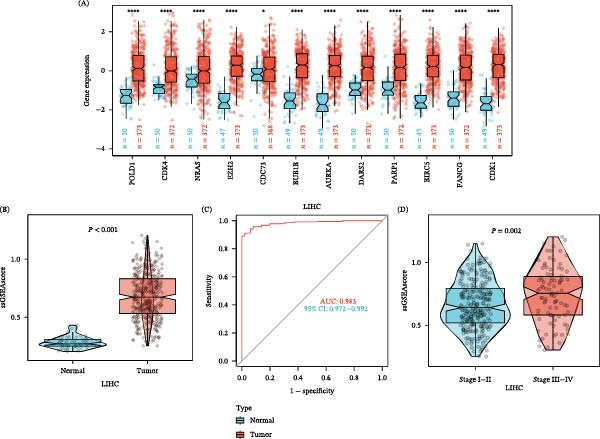
Expression, diagnostic value, and clinical‐stage association of the prognostic gene set. (A) Box plot of expression levels of 12 candidate genes in tumor tissues and adjacent tissues of the TCGA‐LIHC cohort. All genes were significantly upregulated in liver cancer tissues. (B) Violin plot of the distribution of ssGSEA scores of this 12‐gene set in liver cancer and adjacent tissues. The ssGSEA score of liver cancer tissues was significantly higher. (C) ssGSEA score is the receiver operating characteristic curve for differentiating liver cancer from adjacent tissues. The area under the curve (AUC) was 0.983, indicating that it has extremely high diagnostic efficacy. (D) Distribution of ssGSEA scores in liver cancer patients with different TNM stages (Stage I–II vs. Stage III–IV). The scores of patients in the advanced stage (Stage III–IV) were significantly higher than those in the early stage (Stage I–II).

Having confirmed that the risk score is a significant independent prognostic factor, we next evaluated the broader clinical utility of the 12‑gene signature, specifically its diagnostic accuracy in distinguishing tumors from normal tissues and its association with tumor progression (TNM stage). The 12‑gene prognostic signature demonstrated significant diagnostic value and a positive association with advanced tumor stage (Figure 6). As shown in Figure 6, we systematically evaluated the clinical significance of 12 candidate prognostic genes (POLD1, CDK4, NRAS, EZH2, CDC73, BUB1B, AURKA, DARS2, PARP1, BIRC5, FANCG, and CDK1). In the TCGA‐LIHC cohort, the expression of these genes in liver cancer tissues was significantly higher than that in adjacent tissues (Figure 6A). The ssGSEA score further constructed based on this gene set was also significantly increased in tumor tissues (Figure 6B). It is worth noting that this score demonstrates extremely high diagnostic efficacy in differentiating liver cancer from adjacent tissues, with an area under the ROC curve (AUC) as high as 0.983 (95% CI: 0.972–0.992; Figure 6C). In addition, the ssGSEA score of patients in the advanced stage (TNM Stage III–IV) was significantly higher than that of patients in the early stage (Stage I–II; *p* = 0.002), suggesting that this score was positively correlated with the degree of malignant progression of liver cancer (Figure 6D).

### 3.7. Validation of Prognostic Characteristics Based on Single‐Sample GSEA in Multiple Cohorts

**Figure 7:**
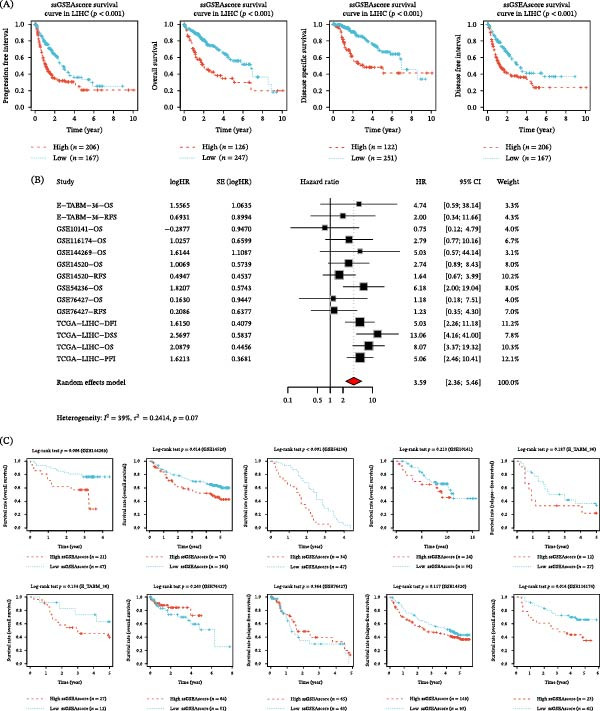
Validation of the ssGSEA‐based prognostic signature across multiple cohorts. (A) The Kaplan–Meier survival curves of patients in the high and low groups based on the ssGSEA score in the TCGA cohort. The overall survival period of patients in the high‐score group was significantly shortened. (B) Meta‐analysis of the prognostic value of ssGSEA score forest plot. The combined results confirmed that it was a consistent independent prognostic factor in different populations. (C) In the GEO external validation cohort, the Kaplan–Meier survival curves of the high and low ssGSEA score groups once again verified its prognostic predictive ability.

The ssGSEA score derived from the 12‑gene set showed high diagnostic accuracy and stage association in the TCGA cohort. To test whether this score generalizes to other patient populations, we performed external validation using independent GEO cohorts and a meta‑analysis of the ssGSEA‑based prognostic value. The prognostic value of the 12‑gene ssGSEA score was validated as universal across multiple independent cohorts (Figure 7). BTo verify the universality of the prognostic characteristics of the 12 genes, we conducted a multicohort survival analysis based on their ssGSEA scores. In the internal TCGA cohort, the OS of patients with high scores was significantly shorter than that of patients with low scores (Figure 7A). Subsequently, a meta‐analysis integrating multiple independent cohorts confirmed that this score was a robust independent prognostic factor (Figure 7B). Finally, in the GEO external validation cohort, this feature also effectively achieved patient risk stratification (Figure 7C). These results collectively indicate that the ssGSEA score based on these 12‐gene characteristics can consistently quantify the biological activity and predict the prognosis of liver cancer patients in different datasets.

### 3.8. Drug Relocation and Molecular Docking Verification Based on Drug Feature Databases

**Figure 8:**
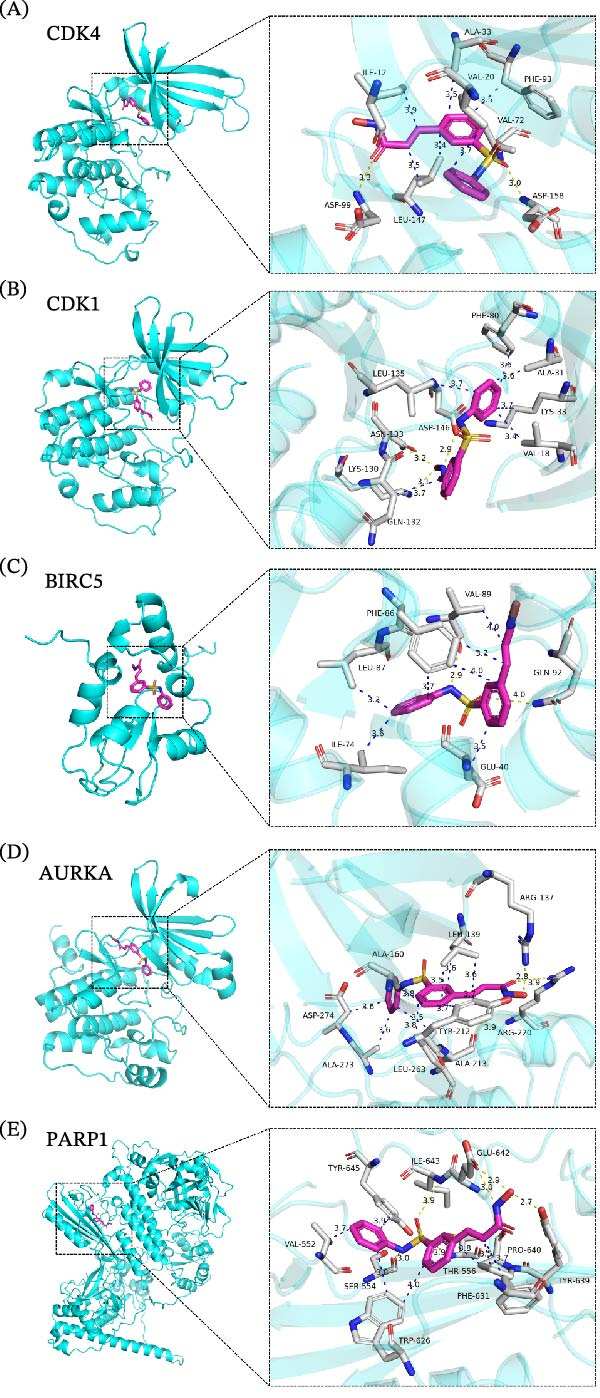
Drug relocation and molecular docking verification based on drug feature databases. (A–E) Molecular docking and binding pattern diagram of the histone deacetylase inhibitor Belinostat with five key prognostic gene protein products (CDK4, EZH2, KRT88, CCNA2, and PCNA). Bellistat exhibits favorable binding free energy and specific molecular interactions (such as hydrogen bonds and hydrophobic interactions) in all targets.

With the prognostic and diagnostic utility of the 12‑gene signature firmly established across multiple cohorts, we next asked whether this signature could be translated into a therapeutic opportunity. Specifically, we sought to identify existing drugs that might reverse the expression pattern of the signature. Thus, we performed drug repositioning analysis using the DSigDB database, followed by molecular docking validation. The FDA–approved HDAC inhibitor belinostat was identified as a potential therapeutic candidate targeting the prognostic signature through drug repositioning analysis and validated by molecular docking (Figure 8). To bridge our prognostic gene signature to actionable therapeutics, we leveraged the DSigDB—a curated resource for linking drugs/compounds to target gene sets for GSEA. DSigDB curates 22,527 gene sets encompassing 17,389 unique compounds and 19,531 genes, enabling systematic integration of drug activity with transcriptomic profiles. We queried DSigDB using our 5‐gene HCC prognostic signature to identify compounds whose target gene sets significantly overlapped with our signature. This analysis prioritized belinostat (CTD:00004313), an FDA–approved HDAC inhibitor, as a top hit. Belinostat exhibited strong signature enrichment (normalized enrichment score, indicating a putative mechanistic association between its pharmacological activity and our prognostic gene network. To validate belinostat’s capacity to modulate core drivers of our signature, we performed molecular docking of belinostat against the protein products of five key prognostic genes: CDK4, EZH2, KRT88, CCNA2, and PCNA (Figure 8A–E). Using AutoDock Vina for simulations, belinostat showed favorable binding affinities and specific interactions—including hydrogen bonds with catalytic residues and hydrophobic contacts with conserved domains—that aligned with established belinostat‐target binding modes. These structural data provide direct evidence for belinostat’s ability to engage core HCC progression drivers encoded by our signature.

## 4. Discussion

HCC remains a lethal malignancy with a complex molecular pathogenesis and limited therapeutic options for advanced stages. In this study, we employed a comprehensive bioinformatics framework integrating differential expression analysis, WGCNA, and CSC biology to identify a robust 12‐gene prognostic signature for HCC [16, 17]. Our findings not only delineate a novel molecular signature with significant diagnostic and prognostic power but also propose a potential repurposing strategy using the HDAC inhibitor belinostat, thereby bridging computational discovery with clinical translation.

In this study, we established three main findings. First, we identified a novel 12‑gene CSC–driven prognostic signature for HCC. Second, this signature demonstrated near‑perfect diagnostic accuracy (AUC = 0.983) in distinguishing HCC from adjacent tissues and was significantly associated with the advanced tumor stage. Third, through computational drug repositioning, we nominated the FDA–approved HDAC inhibitor belinostat as a potential therapeutic candidate targeting this signature. Collectively, these results provide a biologically grounded prognostic tool and a readily translatable treatment strategy for HCC.

The overall logic of our analysis proceeded as a stepwise funnel from broad discovery to focused clinical and therapeutic translation. First, we identified widespread transcriptomic alterations in HCC through differential expression analysis (finding: 2933 DEGs). Second, recognizing that individual genes do not operate in isolation, we applied WGCNA to uncover co‑expression modules functionally linked to clinical traits (finding: the darkslateblue module, *R* = 0.66). Third, to enrich for stemness‑relevant drivers—a key biological axis in HCC aggressiveness—we intersected the disease‑associated module with a CSC gene database, yielding 12 core genes. Fourth, we rigorously compared multiple machine learning algorithms to construct a parsimonious prognostic model, selecting CoxBoost for its superior and consistent performance. Fifth, we validated the model’s prognostic power across independent cohorts via meta‑analysis and demonstrated its diagnostic utility (AUC = 0.983) and association with the advanced TNM stage. Sixth, single‑cell analysis was performed not as an independent discovery but as a mechanistic exploration of where these 12 genes might act within the tumor microenvironment, revealing, for example, DARS2 enrichment in M1 macrophages. Finally, having established a robust clinically relevant signature, we pursued drug repositioning to nominate existing compounds (belinostat) that could potentially target the signature’s core drivers. This sequential logic—from global transcriptome to co‑expression network to stemness‑filtered gene set to prognostic model to clinical validation to cellular resolution to therapeutic nomination—transforms what might otherwise appear as a collection of independent analyses into an integrated and hypothesis‑driven narrative.

The initial transcriptomic landscape revealed profound gene expression alterations in HCC, with 2933 DEGs unequivocally distinguishing tumors from nontumor tissues. This provided a solid foundation for subsequent network analyses. The power of WGCNA lies in its ability to move beyond individual genes to capture biologically meaningful co‐expression modules [18–20]. The identification of the darkslateblue module, strongly correlated with HCC clinical traits and internally validated by the significant MM‐GS relationship, underscores the presence of a coordinated gene network central to HCC pathogenesis. The convergence of this HCC–associated module, a curated CSC gene set, and the DEGs through a Venn intersection was a critical step. It filtered out background noise and pinpointed 12 core genes that likely reside at the intersection of core oncogenic processes, stemness properties, and differential expression. This multimodal filtering enhances the biological plausibility of the final signature, which includes genes like EZH2, CDK4, and AURKA with well‐established roles in cell cycle progression and tumorigenesis [21–23].

The rigorous algorithm comparison established CoxBoost as the optimal method for model construction, highlighting the importance of selecting a modeling strategy that can handle the inherent collinearity and noise in genomic data while effectively performing variable selection [24, 25]. The prognostic robustness of the resulting model was not an artifact of a single dataset but a reproducible phenomenon [26]. Its validity was rigorously confirmed through internal validation (TCGA), external validation (GEO), and a meta‐analysis that statistically aggregated evidence across cohorts, affirming its generalizability across diverse patient populations.

Beyond prognosis, the 12‐gene signature demonstrated a remarkable diagnostic capability. The near‐perfect AUC (0.983) for distinguishing HCC from adjacent tissues suggests its potential utility as a complementary diagnostic biomarker. Furthermore, the significant elevation of the ssGSEA score in advanced TNM stages provides a crucial link between the signature and tumor progression [27, 28]. This implies that the biological processes captured by these genes—potentially encompassing rapid proliferation, stemness, and genomic instability—are not merely present but are amplified as the disease advances [29].

The significant elevation of the ssGSEA score in advanced (Stage III–IV) compared to early (Stage I–II) HCC provides an important mechanistic clue. Rather than being a static property, the stemness‑related transcriptional program captured by our 12‑gene signature appears to be progressively amplified during tumor progression. This suggests that as HCC invades and metastasizes, CSC features—such as self‑renewal, plasticity, and epithelial–mesenchymal transition—become increasingly activated, potentially driving aggressive behavior and therapy resistance. The stage‑dependent increase implies that stemness is not merely a baseline characteristic of HCC but a dynamic axis that escalates with malignant evolution, reinforcing the rationale for targeting this signature even more strongly in advanced disease.

The single‐cell expression analysis added a layer of cellular resolution, hinting at the tumor microenvironment’s role [30–32]. The specific enrichment of DARS2 in M1 macrophages is particularly intriguing. As M1 polarization is coupled with metabolic reprogramming, this finding suggests a potential, though still speculative, involvement of DARS2 in immunometabolic regulation within the HCC niche. This hypothesis warrants future experimental investigation to determine if tumor cells exploit this pathway to modulate anti‐tumor immunity [33, 34]. Perhaps the most translatable finding is the identification and computational validation of belinostat as a putative therapeutic agent [35]. The enrichment in DSigDB and the favorable molecular docking results with key signature proteins (CDK4, EZH2, etc.) provide a strong mechanistic hypothesis. Belinostat, an FDA–approved HDAC inhibitor for peripheral T‐cell lymphoma, could potentially reverse the dysregulated epigenetic and proliferative states driven by our signature. While not yet FDA–approved for HCC, preclinical evidence suggests that HDAC inhibitors can suppress HCC growth, and early‐phase clinical trials are exploring their safety and efficacy in liver cancer, providing a direct translational path for our findings. This offers a compelling rationale for future studies to empirically test belinostat in HCC models characterized by a high 12‐gene signature score [36, 37].

Compared to recently published HCC prognostic models that focus solely on differential expression or immune infiltration, our signature’s key advantage lies in its biological grounding in CSC biology. For instance, while models based on generic proliferation or metabolic genes offer prognostic value, they may not capture the therapy‐resistant and tumor‐initiating subpopulations responsible for relapse, a gap our CSC–driven approach directly addresses. This integration of stemness provides a more mechanistic link to the core drivers of poor outcomes in HCC.

To empirically validate belinostat’s therapeutic potential, future studies should first assess whether belinostat suppresses canonical stemness markers—including CD44, EpCAM, and SOX2—in HCC cell lines characterized by the high expression of our 12‑gene signature. Second, the efficacy of belinostat should be tested in patient‑derived xenograft (PDX) models stratified by the signature score to determine whether high‑signature tumors are preferentially sensitive. Such a stratified preclinical trial would provide direct evidence for signature‑guided patient selection in future clinical studies of belinostat in HCC [38].

Several limitations should be acknowledged. First, the TCGA–LIHC cohort predominantly includes patients with early‐stage, resectable HCC, which may limit the generalizability of our prognostic signature to advanced, unresectable diseases—the very setting where prognostic tools are most urgently needed. Validation in cohorts enriched for stage III/IV HCC is, therefore, essential. Second, while our computational drug repositioning and docking results are promising, they require empirical validation through in vitro and in vivo experiments in HCC models. Third, the DARS2‑macrophage hypothesis, though mechanistically plausible, awaits functional testing. Furthermore, our initial differential expression analysis relied on a single dataset (GSE101685), raising the possibility that the 12‑gene signature may partially reflect dataset‑specific variation rather than universal pan‑HCC biology, although subsequent multicohort validation partially mitigates this concern. Despite these limitations, the multicohort consistency of our signature supports its robustness as a starting point for further clinical and translational investigations.

## 5. Conclusion

Our integrated analysis identifies a novel and robust 12‐gene signature that effectively diagnoses HCC, stratifies patient prognosis, and reflects tumor progression. By linking this signature to the FDA–approved drug belinostat, we provide an actionable road map for translating these bioinformatic insights into potential therapeutic strategies, ultimately moving closer to the goal of personalized oncology for HCC patients. Future studies should explore the efficacy of belinostat, particularly in combination with other modalities like immunotherapy or targeted agents, in HCC patient subgroups defined by a high 12‐gene signature score.

## Author Contributions

**Yang Zi:** conception, analysis, manuscript writing. **Xuehua Yan:** review and edit manuscript. **Ying Zhang** **, Jun Wu, and Jingjing Xie:** study design.

## Funding

This study was supported by the medical and health scientific research projects of the Lanzhou Municipal Health Commission (Project Number A2023028) and the Lanzhou Talent Innovation and Entrepreneurship Project (Project Number 2021‐RC‐119).

## Disclosure

All authors wrote the first draft, read, amended the draft, and finally approved the manuscript.

## Ethics Statement

The authors have nothing to report.

## Conflicts of Interest

The authors declare no conflicts of interest.

## Supporting Information

Additional supporting information can be found online in the Supporting Information section.

## Supporting information


**Supporting Information** Table 1. Full gene lists of cancer stem cell‐related genes, HCC–associated differentially expressed genes, WGCNA key module genes, and their pairwise and three‐way intersections. Cancer stem cell‐related genes were retrieved from GeneCards and differentially expressed genes were identified from the GSE101685 dataset. These gene sets were used for Venn diagram analysis and subsequent prognostic model construction.

## Data Availability

The data that support the findings of this study are available from the corresponding author upon reasonable request.
